# The COVID-19 lockdown as a model of detraining in division 1 college softball players

**DOI:** 10.1186/s13102-024-00836-2

**Published:** 2024-02-10

**Authors:** Giorgio Zeppieri, Cheng-Ju Hung, Marissa Pazik, Michael Moser, Kevin Farmer, Federico Pozzi

**Affiliations:** 1 UF Health Rehabilitation, 3450 Hull Rd, Gainesville, FL 32607 USA; 2 Department of Physical Therapy, University of Florida, 1225 Center Dr, Gainesville, FL 32610 USA; 3 Department of Orthopaedics and Sports Surgery, University of Florida, 3450 Hull Rd, Gainesville, FL 32607 USA; 4 Doctoral Program in Rehabilitation Science, University of Florida, 1225 Center Dr, Gainesville, FL 32610 USA

**Keywords:** Range of motion, Strength, Shoulder, Hip, Preseason

## Abstract

**Background:**

The COVID-19 pandemic interrupted the organized training of softball players, similar to the abrupt cessation of sports participation that can happen after an injury. Thus, the COVID-19 pandemic offers a unique model to study how sudden detraining influences softball players.

**Methods:**

We recruited a sample of convenience of National Collegiate Athletic Association Division 1 softball players. They participated in three data collections: pre-lockdown (Jan 2020, T1), post-lockdown (Sept 2020, T2), and before the 2021 season (Jan 2021, T3). Between T1 and T2, players received an at-home conditioning and throwing program, but compliance was not strictly monitored. Between T2-T3, players resumed formal fall training (team-organized workouts, on-field practice, and within-team scrimmage games). At each time point, we collected bilaterally: 1) shoulder internal rotation (IR) and external rotation (ER) range of motion (ROM); 2) shoulder IR and ER strength; 3) hip IR and ER ROM; and 4) hip abduction and extension strength. We used four independent (2 Sides × 3 Timepoints) MANOVA with repeated measures; we followed up significant MANOVA main effect of time with Sidak posthoc tests for pairwise comparisons between time points.

**Results:**

Fifteen players participated in this study. We found a significant MANOVA main effect of time for shoulder and hip ROM (*p* < 0.01). Between T1-T2, dominant shoulder ER ROM decreased 6.5°, dominant shoulder IR ROM increased 4.3°, and lead hip IR ROM increased 4.4°. Between T2-T3, dominant shoulder ER ROM increased 6.3° and trail hip ER ROM increased 5.9°. We found a significant MANOVA main effect of time for shoulder strength (*p* = 0.03) but not for hip strength (*p* = 0.18). Between T2-T3, non-dominant shoulder IR and ER increased 1.8 kg and 1.5 kg, respectively.

**Conclusion:**

A sudden and prolonged cessation of organized training generated changes in shoulder and hip ROM but affected strength to a lesser extent. The loss of shoulder ER and increased lead hip IR ROM are maladaptive as they are associated with injury in overhead athletes. Resuming team-organized training and scrimmage reversed some (shoulder ER), but not all of these changes. Practitioners should monitor clinical variables regularly and be aware of potential changes due to unexpected and prolonged interruptions in training, such as when players suffer sports-related injuries.

## Introduction

In the spring of 2020, the National Collegiate Athletic Association (NCAA) suspended all collegiate sports to mitigate the effect of the COVID-19 pandemic [1]. The Division 1 college softball season was canceled, and organized training was prohibited [2, 3]. Softball athletes lost access to the university’s weight room, athletic training, and practice facilities [2, 3]. Athletes were also restricted from traditional in-person sport-specific practice and contact with the strength and conditioning, rehabilitation, medical, and coaching staff [4].

Sport-specific training exposes softball players to large repetitive forces across the glenohumeral and lumbo-pelvic-hip joint complexes [5–9]. These repetitive stimuli generate sport-specific adaptations, such as greater shoulder external rotation (ER) range of motion (ROM) and lesser shoulder internal rotation (IR) ROM on the throwing side compared to the contralateral side. Adaptations also occur at the hip with ER and IR ROM decreasing over the course of a season [5, 8, 9]. During the COVID-19 lockdown, athletes were forced to train at home, with limited or no access to equipment and space, causing increased training variability before returning to active participation [4]. It is safe to assume that many athletes could not train at their usual intensity. Studies on muscle stretching showed that ROM across various joints decreases without training stimuli [10–13]. Periods of detraining can lead to a reduction in physiological conditioning [14–16]. Skeletal muscle is highly vulnerable to detraining, with a decrease in strength occurring in less than 1 week [4, 17]. Previous epidemiological studies have demonstrated a correlation between cessation of sports activities (i.e., player lockout), early sports reintegration, and increased injury rates, including throwing-specific injuries (ulna collateral ligament tears in baseball pitchers) [18–20]. However, it is currently unknown how an abrupt stop in sport participation affect clinical measures used to assess athletes’ physical status. This knowledge is important because alterations of ROM and strength can lead to an increased risk of injury [9, 21, 22].

The effects of detraining are not well-known because extended periods of detraining are uncommon in elite athletes. Thus, the COVID-19 pandemic offers a unique model to study how sudden detraining influences softball players. Therefore, this study aimed to examine the impact of the COVID-19 lockdown on shoulder and hip ROM and strength in college softball players. We hypothesized that detraining would generate maladaptive changes of ROM and strength.

## Methods

### Participants

Players from the University of Florida NCAA Division 1 softball team undergo a standardized clinical evaluation every year before starting the competitive season. Players must be healthy and medically cleared for full participation in softball-related activities to participate in the clinical evaluation. Players are excluded from the clinical evaluation if they 1) did not consent to participate and 2) did not recover from a prior injury. Participation in the preseason clinical evaluation occurs through a collaboration between the research team, the athletic department, and the coaching staff. The Institutional Review Board of the University of Florida approved all testing protocols, recording the results of the clinical evaluation and the use of the results for research-related purposes (IRB 201600056). Each participant signed an informed consent before participating in the clinical evaluation. The current study used a sample of convenience that included only athletes of the softball team of the 2020 competitive season. We obtained an amendment to our IRB-approved research protocol (IRB 201600056) that granted permission to perform a clinical evaluation outside of preseason and use these data for research. Each participant signed an informed consent before participating in the clinical evaluation outside of preseason.

### Procedure

Players participated in three data collections: before the start of the 2020 season (T1, January 2020, pre-lockdown), after return to campus (T2, September 2020, post-lockdown), and before the start of the 2021 season (T3, January 2021). The NCAA canceled the 2020 competitive season in March 2020 (between T1 and T2). Players left the University and did not participate in any team-organized strengthening and conditioning activity, sport-specific team training, on-field practice sessions, and games. The team athletic trainer provided players with an at-home conditioning and throwing program. This training was not player-specific. The sudden departure of athletes from campus impacted our ability to set up procedures to measure player’s compliance with home training. Players returned to campus in September 2020 (T2) and resumed standard fall training, which included team-organized softball-specific strengthening and conditioning and on-field practice sessions. Within-team scrimmage games resumed in October 2020.

### Outcomes

Participants filled out a questionnaire to obtain demographic characteristics of age, college year, playing position, and arm dominance (the arm they use to throw a softball). We measured height and weight using a standard scale with a height chart. Upper and lower extremity ROM and strength screening tests were chosen based on relevance and previous utilization for screening overhead athletes. A physical therapist (17 years of clinical experience, an advanced degree in sports clinical specialty, and expertise in collecting clinical outcomes in overhead athletes) collected all the outcome data at each time point. When needed, a sports physical therapy resident assisted the physical therapist by providing stability to body segments during the measurements. We obtained all outcomes bilaterally. At the hip, the trail hip consisted of the hip ipsilateral to the throwing shoulder and the lead hip to the contralateral.

### Range of motion

**Fig. 1:**
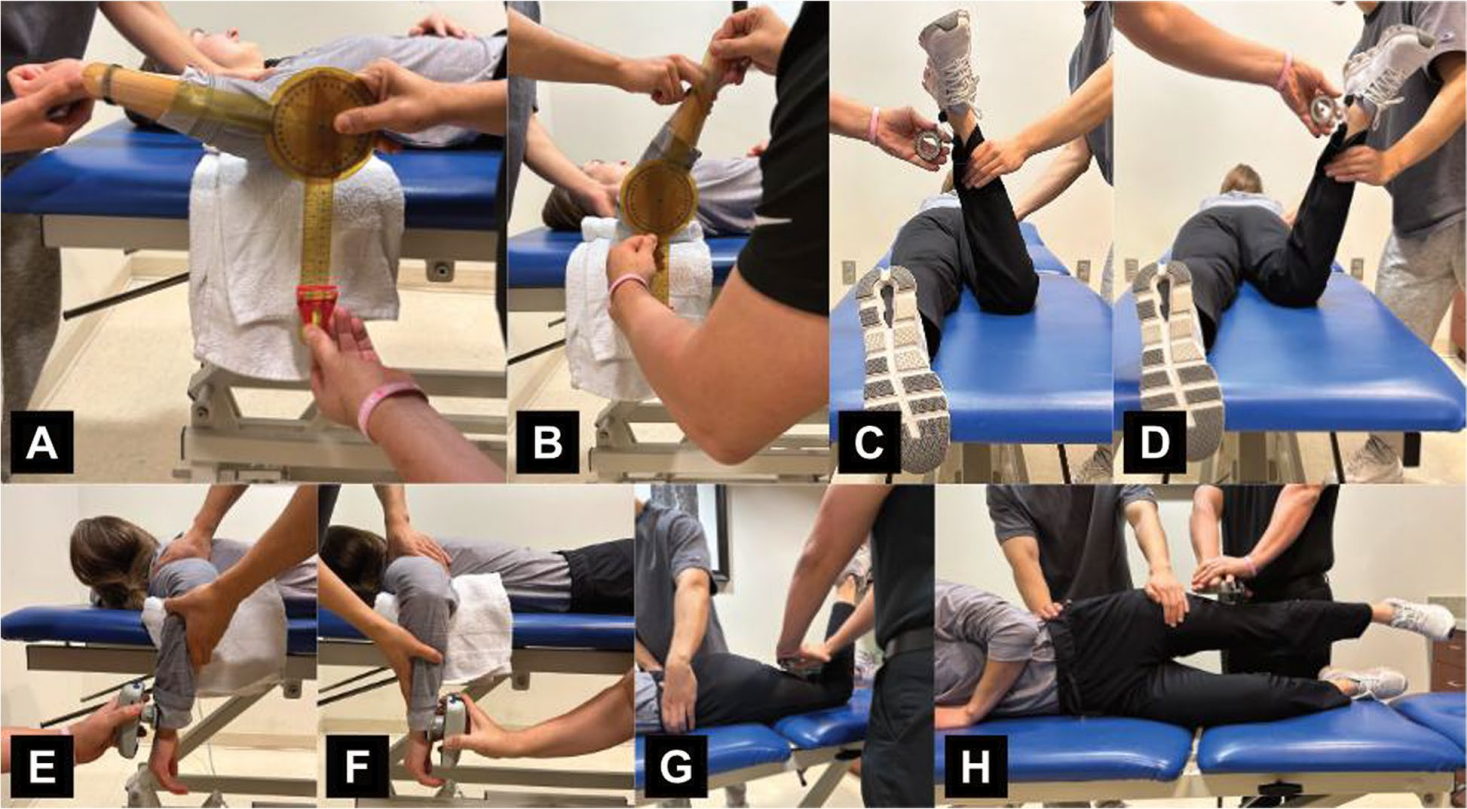
Representative subject illustrating the position used to measure shoulder external (**A**) and internal (**B**) rotation range of motion; hip external (**C**) and internal (**D**) rotation range of motion; shoulder external (**E**) and internal (**F**) rotation strength; and hip extension (**G**) and adduction (**H**) strength

Shoulder ER and IR were measured bilaterally with participants lying supine with the testing shoulder in 90° of abduction and the elbow in 90° of flexion, with a towel roll positioned under the humeral shaft (Fig. 1A, B) [23]. A two-examiner method was used for all IR and ER measurements, with one examiner stabilizing the testing shoulder and one examiner obtaining ROM measurements [23]. One examiner stabilized the shoulder by grasping posteriorly around the scapula and anteriorly around the coracoid with one hand to minimize excess motion while passively moving the participant’s shoulder with their second hand to the end range of glenohumeral joint ER and IR [23]. The second examiner placed the stationary arm of the goniometer perpendicular to the floor, the goniometer’s axis of rotation in line with the participant’s olecranon process, and the parallel arm in line with the participant’s ulna from the axis to the ulnar styloid [7]. This method has been shown to have a good test-retest intratester intraclass correlation (ICC =0.88) [23–26].

Hip ER and IR were measured bilaterally with the participants lying prone with the testing hip at 0° of extension and abduction and knee at 90° of flexion (Fig. 1C, D) [27–29]. A two-examiner method was used for all IR and ER measurements, with one examiner stabilizing the pelvis and one examiner obtaining ROM measurements [27–29]. One examiner stabilized the pelvis with one hand to minimize excess motion while passively moving the participant’s hip with the second hand to end range of coxofemoral joint ER and IR [27, 28]. The second examiner placed the bubble inclinometer proximally on the participant’s medial malleolus. This method has excellent test-retest reliability (ICC = 0.98) [25, 27–29].

### Strength

We used a microFET 2 digital handheld dynamometer (Hoggan Health Industries, Salt Lake City, Utah) to measure shoulder and hip isometric strength. The microFET 2 has a certificate of calibration and documented accuracy of up to 1%. Participants performed one repetition for each strength test. As described below, the examiner positioned the dynamometer and acted as a fixed resistance point. Participants were instructed to push against the resistance provided by the examiner gradually to reach a maximum force and maintain the maximal force while the examiner counted to 5 seconds. The examiner matched the force produced by each participant to keep the body segment steady (i.e., isometric contraction).

Shoulder ER and IR strength were measured with participants lying prone on a treatment plinth to assist in stabilizing the trunk during the exam (Fig. 1E, F). The shoulder was positioned in neutral rotation and 90° of abduction and the elbow was placed in 90° of flexion, with a towel roll positioned under the humeral shaft to ensure proper horizontal alignment. One examiner stood adjacent to the testing side shoulder to stabilize the scapula and maintain the humerus in the proper testing position. The second examiner placed the dynamometer on the dorsal side of the forearm for ER and the palmar side of the forearm for IR proximal to the wrist and between the radius and the ulna styloid. The participant was asked to rotate externally (for ER strength) or internally (for IR strength) against the dynamometer’s force pad. This method has excellent relative reliability (ICC3,1) values with 95% interclass correlation for dominant and non-dominant shoulder ER (ICC = 0.95–0.96) and dominant and non-dominant IR (ICC = 0.94–0.97) [25, 30, 31].

Hip extension (EXT) strength was measured with the participant prone with the knee flexed to 90° (Fig. 1G) [28, 32]. Prior to testing, each hip was passively extended to ensure the absence of hip flexor tightness, which could alter the test results [28]. One examiner was positioned ipsilateral to the testing hip and stabilized the participant’s pelvis at the lumbar spine [28, 33]. The second examiner placed the dynamometer on the posterior aspect of the thigh, proximal to the popliteal fossa [28]. This method has good interrater reliability (ICC2,1), ICC = 0.88 [25, 33].

Hip abduction (ABD) strength was measured with participants in a side-lying position, with their testing hips facing up (Fig. 1H) [28, 32]. The tested hip was slightly extended beyond the midline of the pelvis, while the non-tested lower extremity was positioned at 40° of hip flexion and 90° of knee flexion [30, 33, 34]. The second examiner stabilized the pelvis along the lumber spine and anterior iliac spine. The dynamometer was placed proximal to the lateral condyle. Participants were asked to abduct the hip and push against the dynamometer’s force pad [30, 33–35]. This method has good intrarater reliability (ICC3,1) ICC = .82 and interrater reliability (ICC2,1) ICC = 0.81 [25, 27, 33].

### Statistical analysis

Based on the available participants (*N* = 15) and alpha of 0.05, we calculated the effect size that we could detect with 80% power with a paired t-test using G*Power (version 3.1) [36, 37]. Based on published standard deviations [8, 35], the calculated effect size (0.6) translates to a mean difference of 6.2° for shoulder ER ROM, 5.5° for shoulder IR ROM, 4.9° for hip ER ROM, 4.4° for hip IR ROM, 1.9 kg from shoulder ER strength, 1.7Kg for shoulder IR strength, and 2.3 kg for hip abduction and extension strength.

All analyses were performed using SPSS Version 27 (IBM Corporation, Armonk, NY). Missing data was low (one participant’s hip strength at T1 and one participant’s shoulder strength at T2). We imputed data at T1 using the next observation carried backward and at T2 using the last observation carried forward. We used four independent 2 (Side, dominant and non-dominant side for the upper extremity and lead and trail leg for the lower extremity) by 3 (Timepoints) repeated-measures multivariate analysis of variance (MANOVA) models, one for shoulder ROM, one for hip ROM, one for shoulder strength, and one for hip strength. The main scope of this work was to understand the effect of time on clinical screening variables; thus, we followed up significant MANOVAs’ main effect of time with Sidak posthoc tests for pairwise comparisons between time points (T1 vs. T2 and T2 vs. T3). The alpha level was set at 0.05. We calculated the effect size (Cohen’s *d*) of the mean differences between time points. The effect size was interpreted as follows: d = 0.2, small; d = 0.5, medium; d = 0.8, large [38].

## Results

### Participants

Of the 23 players on the 2020 team, 22 consented to participate in the preseason screening. We excluded players in their last year of college sports eligibility (*N* = 6) because they would leave the team at the end of the 2020 academic year. Our sample included 15 female players because softball is a women only collegiate sport in the United States (Table 1).

**Table 1:** Demographic characteristics of the recruited sample (*N* = 15) measured at first encounter (January 2020). Data are presented as mean ± standard deviation unless otherwise indicated

Female sex, n (%)	15 (100%)^a^
Age, years	19.6 ± 1.0
Height, m	1.7 ± 0.1
Weight, Kg	77.6 ± 10.0
BMI, kg/m^2^	27.0 ± 3.3
Right hand dominant, n (%)	13 (86.7)
Position, n (%)	
Pitcher	4 (26.7)
Outfield	4 (26.7)
Infield	7 (46.7)
College year, n (%)	
1st year	2 (13.3)
2nd year	9 (60.0)
3rd year	4 (26.7)

^a^ Softball is a women only collegiate sport in the United States

### Range of motion

**Fig. 2:**
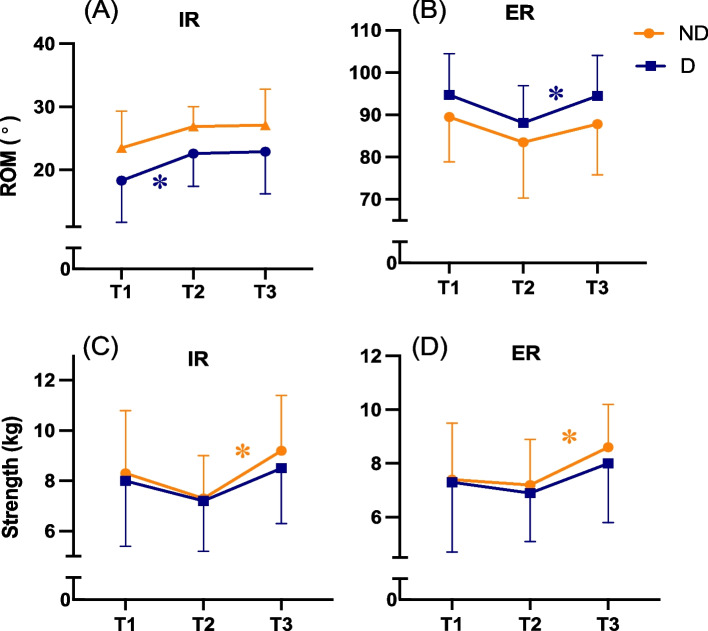
Internal (panel **A**) and external (panel **B**) rotation range of motion & internal (panel **C**) and external (panel **D**) rotation strength for the dominant (D) and non-dominant (ND) shoulder at the three time points of the study. Abbreviations: T1, January 2020; T2, September 2020; T3, January 2021; IR, internal rotation; ER, external rotation.*, Posthoc comparison with Sidak adjustment for multiple comparison *p* < 0.05

**Fig. 3:**
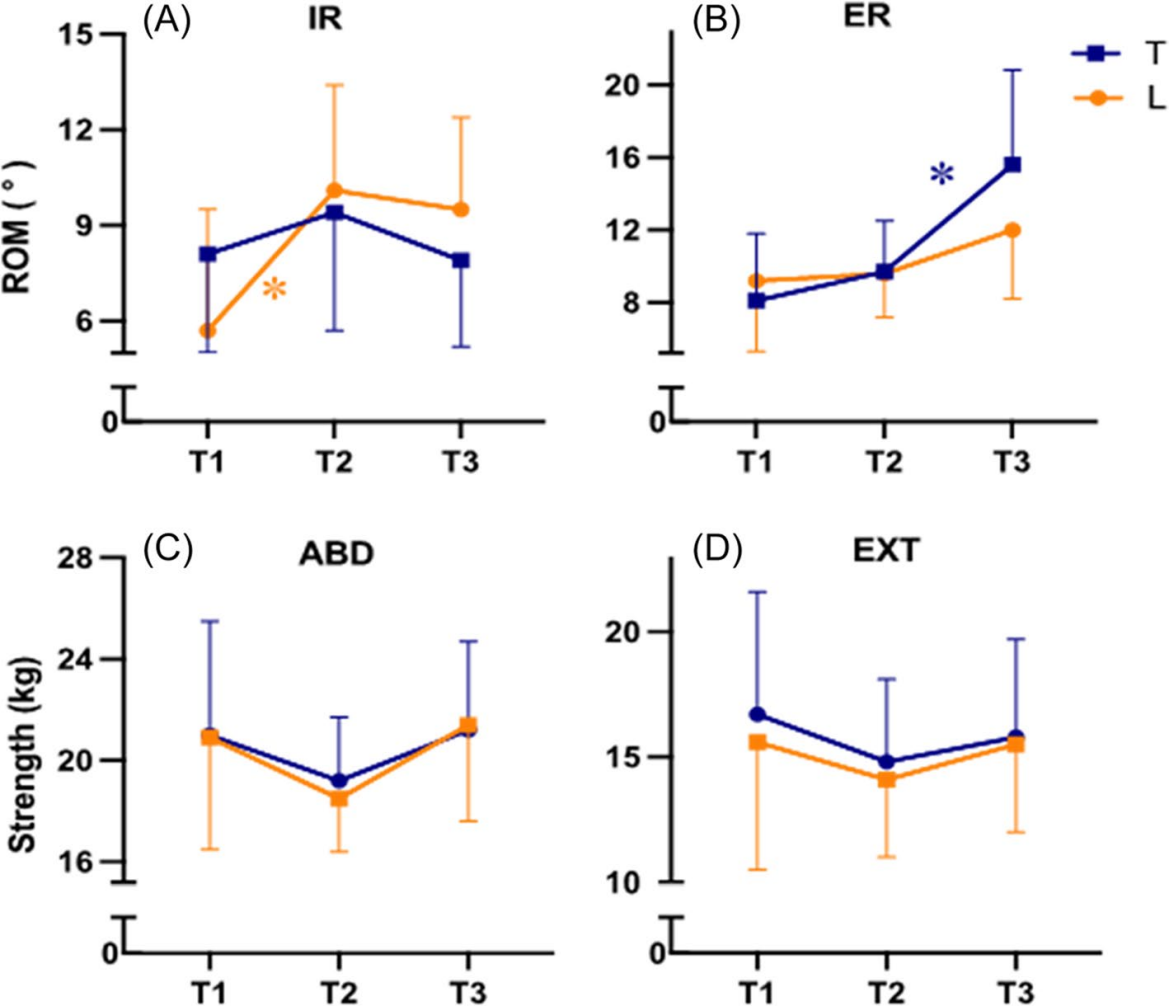
Internal (panel **A**) and external (panel **B**) rotation range of motion & abduction (panel **C**) and extension (panel **D**) strength for the lead (L) and trail (T) hip at the three time points of the study. Abbreviations: T1, January 2020; T2, September 2020; T3, January 2021; IR, internal rotation; ER, external rotation; ABD, abduction; EXT, extension.*, Posthoc comparison with Sidak adjustment for multiple comparison *p* < 0.05

A significant MANOVA main effect of time was found for shoulder and hip ROM (*p* < 0.01). Dominant shoulder IR ROM increased 4.3° from T1 to T2 (*p* = 0.02, Table 2 & Fig. 2A), whereas dominant shoulder ER ROM decreased 6.5° from T1 to T2 (*p* = 0.05, Table 2 & Fig. 2B), but increased 6.3° from T2 to T3 (*p* = 0.03). Lead hip IR ROM increased by 4.4° from T1 to T2 (*p* = 0.03, Table 2 & Fig. 3A), and hip ER ROM increased by 5.9° from T2 to T3 (*p* < 0.01, Table 2 & Fig. 3B).

**Table 2:** Mean change score of shoulder & hip range of motion & strength between time points

		T1	T2	T3	Change from T1 to T2^a^	Change from T2 to T3^b^	*p* value^c^
**Shoulder range of motion, °**							< 0.01
**IR**	**D**	18.3 ± 6.6	22.6 ± 5.2	22.9 ± 6.7	4.3 (0.8, 7.9)^d^ *d* = 0.9	0.3 (−5.8, 6.4) *d* < 0.1	
	**D**	18.3 ± 6.6	22.6 ± 5.2	22.9 ± 6.7	4.3 (0.8, 7.9)^d^ *d* = 0.9	0.3 (−5.8, 6.4) *d* < 0.1	
**ER**	**D**	94.7 ± 9.8	88.1 ± 8.8	94.5 ± 9.6	−6.5 (− 13.1, < 0.1)^d^ *d* = 0.7	6.3 (0.5, 12.2)^d^ *d* = 0.8	
	**ND**	89.5 ± 10.6	83.5 ± 13.2	87.8 ± 12.0	−6.0 (− 12.2, 0.2) *d* = 0.7	4.3 (− 2.2, 10.9) *d* = 0.5	
**Hip range of motion, °**							< 0.01
**IR**	**T**	8.1 ± 3.1	9.4 ± 3.7	7.9 ± 2.7	1.3 (− 2.0, 4.7) *d* = 0.3	− 1.4 (− 4.4, 1.5) *d* = 0.4	
	**L**	5.7 ± 3.8	10.1 ± 3.3	9.5 ± 2.9	4.4 (0.5, 8.3)^d^ *d* = 0.8	−.6 (− 3.2, 2.0) *d* = 0.2	
**ER**	**T**	8.1 ± 3.7	9.7 ± 2.8	15.6 ± 5.2	1.6 (− 2.2, 5.4) *d* = 0.3	5.9 (1.9, 9.8)^d^ *d* = 1.0	
	**L**	9.2 ± 3.9	9.6 ± 2.4	12.0 ± 3.8	0.4 (− 3.1, 2.3) *d* = 0.1	2.4 (− 1.5, 6.3) *d* = 0.4	
**Shoulder strength, kg**							0.03
**IR**	**D**	8.0 ± 2.6	7.2 ± 2.0	8.5 ± 2.2	− 0.8 (− 2.6, 1.0) *d* = 0.3	1.3 (− 0.2, 2.9) *d* = 0.6	
	**ND**	8.3 ± 2.5	7.3 ± 1.7	9.2 ± 2.2	− 1.0 (− 2.5, 0.5) *d* = 0.5	1.8 (0.6, 3.0)^d^ *d* = 1.1	
**ER**	**D**	7.3 ± 2.6	6.9 ± 1.8	8.0 ± 2.2	− 0.4 (− 2.2, 1.4) *d* = 0.1	1.1 (− 0.2, 2.4) *d* = 0.6	
	**ND**	7.4 ± 2.1	7.2 ± 1.7	8.6 ± 1.6	− 0.2 (− 1.7, 1.4) *d* = 0.1	1.5 (0.6, 2.3)^d^ *d* = 1.1	
**Hip strength, kg**							0.18
**ABD**	**T**	21.0 ± 4.5	19.2 ± 2.5	21.2 ± 3.5	− 1.8 (− 5.2, 1.6) *d* = 0.4	2.0 (− 0.1, 4.1) *d* = 0.7	
	**L**	20.9 ± 4.4	18.5 ± 2.1	21.4 ± 3.8	−2.5 (− 6.0, 1.1) *d* = 0.5	2.9 (< 0.1, 5.7) *d* = 0.7	
**EXT**	**T**	16.7 ± 4.9	14.8 ± 3.3	15.8 ± 3.9	−1.9 (− 5.9, 2.0) *d* = 0.3	(−1.6, 3.7) *d* = 0.3	
	**L**	15.6 ± 5.1	14.1 ± 3.1	15.5 ± 3.5	−1.6 (− 5.6, 2.4) *d* = 0.3	1.5 (− 1.1, 4.1) *d* = 0.4	

*T1* January 2020: *T2* September 2020: *T3* January 2021: *IR* internal rotation: *ER* external rotation: *ABD* abductor: *EXT* extensor: *D* dominant side: *ND* non-dominant side: *T* trail side: *L* lead side.

^a^Calculated as T1 – T2. Presented as mean difference (95% confidence interval), effect size (Cohen *d*)

^b^Calculated as T2 – T3. Presented as mean difference (95% confidence interval), effect size (Cohen *d*)

^c^MANOVA time main effect

^d^Sidak adjustment for multiple comparisons, *p* < 0.05

### Strength

A significant MANOVA main effect of time was found for shoulder strength (*p* = 0.03), but not for hip strength (*p* = 0.18, Fig. 3C and D). Non-dominant shoulder IR and ER increased 1.8 kg (*p* < 0.01) and 1.5 kg (*p* = 0.02) from T2 to T3, respectively (Table 2 & Fig. 2C and D).

## Discussion

We used the COVID-19 lockdown as a model to investigate detraining in a prospective cohort of college softball players. A sudden cessation of organized training increased shoulder IR ROM and lead hip IR ROM, and decreased shoulder ER ROM, but affected shoulder and hip strength to a lesser extent. The loss of shoulder ER and increase in lead hip IR ROM are considered maladaptive as they have been associated with injury in overhead athletes [21, 22, 39–41]. Resuming team-organized training, practice, and games reversed some (shoulder ER ROM), but not all of these changes. These results provide insight to facilitate clinical decisions when returning a softball athlete to competitive play after extended time loss due to injury. These findings are important, considering that not all collegiate softball athletes have access to a state-of-the-art care team and facilities for injury management and recovery.

### Shoulder ROM

Shoulder IR ROM increased, while shoulder ER ROM decreased after 5 months of COVID-19 lockdown. These results are opposite of the adaptations seen in throwing athletes (increased dominant ER and decreased IR) [5, 9, 42] and are likely due to reduced training volume, limited access to training facilities, and lack of participation in organized team practice and throwing programs. The loss of shoulder ER ROM may be meaningful because the minimal detectible change for this outcome varies between 4.5 and 7.3° [43, 44]. The loss of sport-specific training adaptions is analogous to the deficits in clinical measures after injury. For example, following shoulder instability surgery, patients present with decreased ROM and strength [17]. The loss of shoulder ROM can lead to decreased functional and sport-specific abilities [17]. Failing to adequately address these deficits, coupled with early return to sports, can lead to suboptimal performance and increased throwing-related injury risk [18, 19]. Evidence of sport-related injuries increasing due to insufficient sports preparation has been shown in Major League Baseball (MLB) following COVID and in the National Football League (NFL) after the 2011 owner/player lockout [19, 45].

Dominant shoulder ER ROM increased after the resumption of organized training. This finding is consistent with previous literature reporting increases in shoulder ER ROM with increases in throwing and training volume in overhead athletes [5, 9, 22, 41, 42]. However, we did not observe a decrease of dominant shoulder IR ROM after the resumption of training, which may indicate that a restricted shoulder IR ROM may be soft tissue related in softball players. However, this interpretation of our findings cannot be entirely determined by the measures and analysis used in our study. These findings contrast previous research showing that a bony deformation (humeral torsion) underlies restricted shoulder IR in baseball players [5, 9, 41, 42]. Our results are consistent with Guy et al., who demonstrated increased shoulder IR ROM in college softball players throughout a season [8]. Dwelly et al. [46] also did not observe changes in shoulder IR ROM throughout a season; however, their sample included both male baseball and female softball players of all positions in their analysis. Furthermore, Ellenbecker and Roetert [47] showed that shoulder IR ROM did not change in female college tennis players throughout the season. Therefore, it is plausible that softball player shoulder IR ROM may not respond similarly to other overhead populations due to the specific softball-related demands (i.e., length of throws, field size). Conversely, a three-month team-organized return to sport program may provide enough training stimuli to return shoulder ER ROM to baseline values effectively. These results may offer a suggestive timeframe when returning softball players to competitive play after extended time loss due to injury.

### Hip ROM

Trail and lead hip ROM decreases throughout a season in professional baseball players [48], and collegiate baseball and softball pitchers [8, 28]. Our findings demonstrated that lead hip IR ROM increased after 5 months of COVID-19 lockdown. Lead hip IR ROM is likely more sensitive to training and workload changes than IR in the trail leg [48] because it aids shoulder deceleration associated with throwing velocity or distances [49]. Thus, the absence of competitive throwing or formal long toss programs may reduce the demands on the lead hip.

Trail hip ER ROM increased significantly once training resumed. This result contradicts previous studies that reported a decrease in trail hip ER ROM throughout a season, after games, and increased throwing volume in overhead athletes [8, 28, 48, 49]. It is possible that although training resumed in September 2020, the training loads were still limited. Fall seasons in college softball are less demanding compared to spring seasons based on training time, games played, and the intensity of competition. Furthermore, the 2020 fall season was further limited in training time due to COVID protocols. Throwing workload is likely a critical variable that impacts hip ROM [48]. Thus, trail hip ER ROM may be less responsive to changes in a mixed cohort of position players and pitchers that mostly throw using a windmill motion.

### Shoulder and hip strength

Inconsistent offseason training, lack of access to equipment, and mandatory isolation after exposure to COVID-19 can impact training loads and seasonal preparation. However, to our surprise, we did not measure a significant decrease in shoulder and hip strength after the COVID-19 lockdown [50, 51]. The team athletic trainer provided participants with an at-home strengthening and conditioning program. Although we did not collect training compliance, we speculate that home strengthening and conditioning programs are easier to execute during isolation than throwing programs. Thus, they may provide enough stimuli to maintain isometric strength performance. It is possible that shoulder strength may be less sensitive to the reduction of sport-specific workload (i.e., throwing) compared to shoulder ROM. Strength appears to return to baseline values after 3 months of organized training. This finding is consistent with previous work: trained athletes can return to baseline strength even after 20 weeks of detraining [52, 53] and reach previous levels of one-repetition maximum strength after 8 weeks of retraining [54].

### Limitation

This study has limitations that should be considered when interpreting the results. COVID-19 created an abrupt cessation of sporting activities. This unplanned occurrence impacted our ability to objectively quantify detraining, control the lockdown training program, and strictly measure compliance. We obtained data from one softball team and the low number of participants prevented stratified analysis based on position (pitchers vs. position players). Our results are limited to NCAA Division 1 college softball players (female only) and cannot be generalized to different sex, other overhead/throwing athletes, or softball players of different ages and skill levels. The study timeframe between T1 and T2 included 2 months of regular team activities and approximately 6 months of at-home training during the lockdown. We cannot attribute our results solely to the COVID-19 lockdown. The absence of regular training was likely the main contributor to the observed changes in our outcomes, but other factors could have played a role during that time.

## Conclusion

A sudden and prolonged detraining caused shoulder and hip ROM changes in college softball players. Loss of dominant shoulder ER and increase in lead hip IR ROM are considered maladaptive as they have been associated with injury in overhead athletes. A three-month team-organized return to sport program may provide enough training stimuli to return shoulder ER ROM to baseline values. Practitioners should monitor clinical variables regularly and be aware of potential changes due to unexpected and prolonged interruptions in training, such as when players suffer sports-related injuries.

## Data Availability

The dataset of the current study is available from the corresponding author upon reasonable request.
